# Zerumbone reduces proliferation of HCT116 colon cancer cells by inhibition of TNF-alpha

**DOI:** 10.1038/s41598-018-22362-1

**Published:** 2018-03-06

**Authors:** Salam Pradeep Singh, Khumukcham Nongalleima, Ningthoujam Indrajit Singh, Pradip Doley, Chingakham Brajakisor Singh, Thiyam Ramsing Singh, Dinabandhu Sahoo

**Affiliations:** 1 Institute of Bioresources and Sustainable Development, Takyelpat, Imphal, 795001 Manipur India; 2 Department of Biotechnology, Manipur University, Canchipur, 795003 Manipur India

## Abstract

Zerumbone is a known anti-cancer herbal compound. However, the actual protein target is not fully understood or known. This investigation focus on the association of zerumbone in HCT116 colon cancer cell proliferation and its link with TNF-alpha. The study shows that with the increasing concentration of zerumbone, there was a reduction of HCT116 cells proliferation based on the cell line study and hence higher TNF-alpha inhibition based on the TNF-alpha assay. The study also emphasizes on the computational aspect by investigating the molecular docking analysis of zerumbone against TNF-alpha. The docked complex was further validated using molecular dynamics simulation studies. The docking analysis observed that alpha-beta unsaturated carbonyl scaffold is an important moiety for the anti-cancer activity of zerumbone. Furthermore, the DFT analysis also confirms the reactivity nature of zerumbone based on the frontier molecular orbital analysis.

## Introduction

Colorectal or colon cancers occur because of unhealthy lifestyle and old age and in some cases because of genetic factors^1,2^. It is also the third-leading cause of cancer-related deaths in US^3^. The risk factors include smoking, obesity, unhealthy diet etc^4^. The risk factors also include Crohn’s disease and ulcerative colitis which are part of inflammatory bowel disease^5^.

On the other hand, zerumbone has been tested for various anti-cancer activities including colon cancer. Zerumbone is a cyclic sesquiterpene extracted from the rhizomes of *Zingiber zerumbet* Smith^6^. Its rhizomes have been used traditionally for folk medicine and pain relief ^7^.

Moreover, recent study on zerumbone observed that zerumbone possess unique and potent anticancer activity against colon cancers cells^8^. It has also shown to inhibit human colonic adeno-carcinoma cell proliferation^9^. The present investigation deals with the anti-cancer activity of zerumbone against HCT116 cells (colon cancer cell line) and its subsequent role in TNF-alpha inhibition in cancer cell proliferation. The linkage of zerumbone inhibiting TNF-alpha and its role in the proliferation of colon cancer cell has never been reported or study.

The TNF gene, in both the mouse and human, is positioned in the major histocompatibility complex, close to the LT3 gene^10,11^. Because of this chromosomal arrangement and their sequence homology TNF has also become known as TNF-alpha^12^. Although the cytotoxic activity of TNF has been well documented, the mechanism of TNF-induced lysis is not well understood^13^. In fact, the TNF is cytotoxic for many tumoral cell lines, whereas normal cells generally are considered resistant to this action^14^. In F17 cells, TNF treatment induced a classical form of apoptosis, while TNF induced a necrotic form of cell death in L-M cells^13^. The TNF-alpha is also a pleiotropic cytokine that activates T-cells via both TNF-Receptor (R-I and R-II) and mediates both apoptotic and survival signals^15–17^. The TNF-alpha mediates its biological functions predominantly via TNFR-I. Following binding of TNF-alpha to TNFR-I, the TNFR-associated death domain (TRADD) is recruited to TNFR-I forming a platform for downstream signaling^18,19^. Then, the TNRF-associated factor 2 (TRAF2) and receptor-interacting protein kinase 1 (RIPK1) are recruited to TRADD forming a signaling complex. The TRADD also recruits fas-associated death domain (FADD), which initiates activation of apical caspases resulting in activation of effector caspases, and apoptosis^20,21^. However, signaling through TNFR1 might also induce apoptosis in a caspase-independent manner^22^.

The present study involves morphological changes of HCT116 Cells treated with zerumbone in various concentrations and the supernatant was utilize for the inhibition of TNF-alpha using Mouse TNF-alpha ELISA kit. The study was cross examined using molecular docking techniques with zerumbone against TNF-alpha enzyme (PDB ID 5MU8) and the stability of the protein-ligand docked complex confirmed by molecular dynamics simulation. Furthermore, the frontier molecular orbital study was carried out to understand the HOMO and LUMO energies of zerumbone that might contribute in the reactivity of the molecule at the active site of the enzyme.

## Materials/Experimental

### Cell line study and TNF-alpha assay

*Z. zerumbet* sample was collected from Moreh, Manipur, India (94.34217° E and 24.35172° N) and given the institutional accession number as IBSD/Z-42-23. Zerumbone was extracted from the oil extract of the fresh rhizomes and identified by FT-IR, Proton and 13 C NMR and mass spectrophotometer.

HCT116 cells were purchased from ATCC (Manassas, VA) and cultured in McCoy’s 5 A medium supplemented with 10% FBS having 100 units/ml penicillin and 100 μg/ml streptomycin solution in a humidified atmosphere of 5% CO2 at 37 °C. Cells were seeded in 24-well plate with a density of 5 × 10^4^ cells/well. To adhere the cells it was incubated for 16 h and treated with cisplatin (25 µg/µL) (Sigma Aldrich, USA) and zerumbone in various concentration (5, 10 and 20 μM) for 24 h. The cells from the media was collected and centrifuged for 15 min at 5000 rpm and the supernatant was utilize for the TNF-alpha assay using Mouse TNF-alpha ELISA kit (Millipore, USA) as per the standard protocol of the manufacturer.

The release of TNF-alpha level in the HCT116 cells was examined for the zerumbone concentrations (5, 10 and 20 μM). Cisplatin, a known anti-cancer drug was used as positive control and the untreated cells were used as the negative control.

The results were expressed as the mean ± SEM for three replicates. The analysis was done in GraphPad Prism 7 (https://www.graphpad.com/). All the graphs and figures were prepared using the mean values in GraphPad Prism 7.

#### Flexible docking

Human TNF-alpha (PDB ID: 5MU8) was retrieved from Protein Data Bank (http://www.rcsb.org/pdb/explore.do?structureId = 5mu8) and loaded in Molegro Virtual Docker 6.02 (MVD). The binding site was predicted and set at the coordinates (X: −21.51, Y: 18.21, Z: −1.82) (Fig. S1 of supplementary material). The residues surrounding the active site region (Glu 23, Val 150, Phe 144, Leu 26, Tyr 141, Asn 19). On the other hand the 3D conformer of zerumbone and α-humulene was retrieved from PubChem and optimized using Gaussian 09 and loaded in the MVD. MolDock Grid Score was set as the scoring function with a grid resolution of 0.30 Å. Additional evaluation for Internal ES (ElectroStatic) and Internal Hbond (Hydrogen bond) were also set for the simulation. The algorithm was set at a maximum iteration of 1,500 having a maximum evolution size of 50. The docking engine was run for 1000 times for better accuracy and minimum of 1000 poses^23^. The best pose of zerumbone and α-humulene was considered for the ligand–protein interaction analysis and MD simulation studies.

### Binding affinity prediction

The binding affinity of the best docked pose of zerumbone and α-humulene was calculated using the MLR equation implemented in Molegro Data Modeller (Affinity = −19.0155 * C0 + 3.3813 * ′′Cofactor (hbond)′′− 0.594128 * Csp2 − 0.061912 * ′′E-Intra (vdw)′′ + 0.464056 * HBond + 0.483368 * HeavyAtoms + 0.953672 * ′′E-Solvation′′ + 3.0229 * Nplus − 3.9493 * halogen − 1.00763 * N − 3.10696 * OS + 1.61426 * OH).

### Molecular dynamics simulation studies

MD simulations were carried out for 5MU8, 5MU8-zerumbone and 5MU8-α-humulene docked complex. MD simulation was carried out using GROMACS version 5.1.2 operated on Ubuntu 16.04. The topology file for PDB ID 5MU8 was processed with OPLS-AA/L force field while 5MU8-zerumbone complex was processed with GROMOS96 43a1^24^. The system was solvated using the equilibrated 3-point solvent model (spc216) and ions were added. The assembled system was relaxed by energy minimization and equilibrated using the NVT and NPT scale for 100 ps. The well-equilibrated was finally run for 30 ns MD production. The generated trajectory file was analyzed for RMSD, RMSF, SASA, Hbond and Radius of gyration.

### MM-PBSA calculations

To adhere the molecular docking simulation results and MD simulation trajectory analysis, the binding free-energy (ΔG_bind_) calculations were carried out for the 30 ns simulated complexes of the 5MU8-Zerumbone complex and 5MU8-α-humulene complex using g_mmpbsa (http://rashmikumari.github.io/g_mmpbsa/). The Molecular Mechanics Poisson-Boltzmann Surface Area (MM-PBSA) estimates the free energy interactions as defined by Kollman *et al*.^25^. The equation of the ΔG_bind_ is represented by the following equation

1 $${{\rm{\Delta }}{\rm{G}}}_{{\rm{bind}}}={{\rm{G}}}_{{\rm{complex}}}\,-({{\rm{G}}}_{{\rm{receptor}}}+{{\rm{G}}}_{{\rm{ligand}}})$$

The binding free-energy of the 5MU8-Zerumbone and 5MU8-α-humulene complexes were analyzed by taking 100 snapshots at an interval of 100 ps from the last 30 ns MD simulation during its equilibrium phase using g_mmpbsa and the final average binding energy was obtained by executing the MmPbSaStat.py python script.

### DFT Calculations

The conformation of the best docking pose of zerumbone and α-humulene was exported and DFT calculations were carried out using Gaussian 09^26^. Theoretical calculations were carried out at Ground State using DFT/B3LYP/LanL2DLZ. The guess method was set as extended Huckel with mix HOMO and LUMO orbital. Population analysis was performed for Merz-Kollman (ESP) charges for full natural bond order (NBO) analysis. The molecular orbital energies were considered for calculating the band energy gap ΔE_*LUMO-HOMO*_.

### Physicochemical properties and ADME-Toxicity calculation

The Physicochemical property and ADME-Toxicity parameters of zerumbone and α-humulene were computed using SwissADME^27^ to estimate the pharmacokinetics and their medicinal chemistry property. These parameters were calculated because drug development and discovery process involve assessment of absorption, distribution, metabolism and excretion and their toxic effects^27^. The newly introduced methods such as BOILED-Egg diagram, iLOGP and Bioavailability Radar map was also calculated for zerumbone and α-humulene. Lastly, LD_50_ and health effect analysis was carried out using ACD Ilab 2.0 (https://ilab.acdlabs.com/iLab2/) and comparative a chart was plotted to compare the two molecules.

### Compliance with Ethical Standards

This article does not contain any studies with human participants or animals performed by any of the authors.

## Results and Discussion

**Figure 1:**
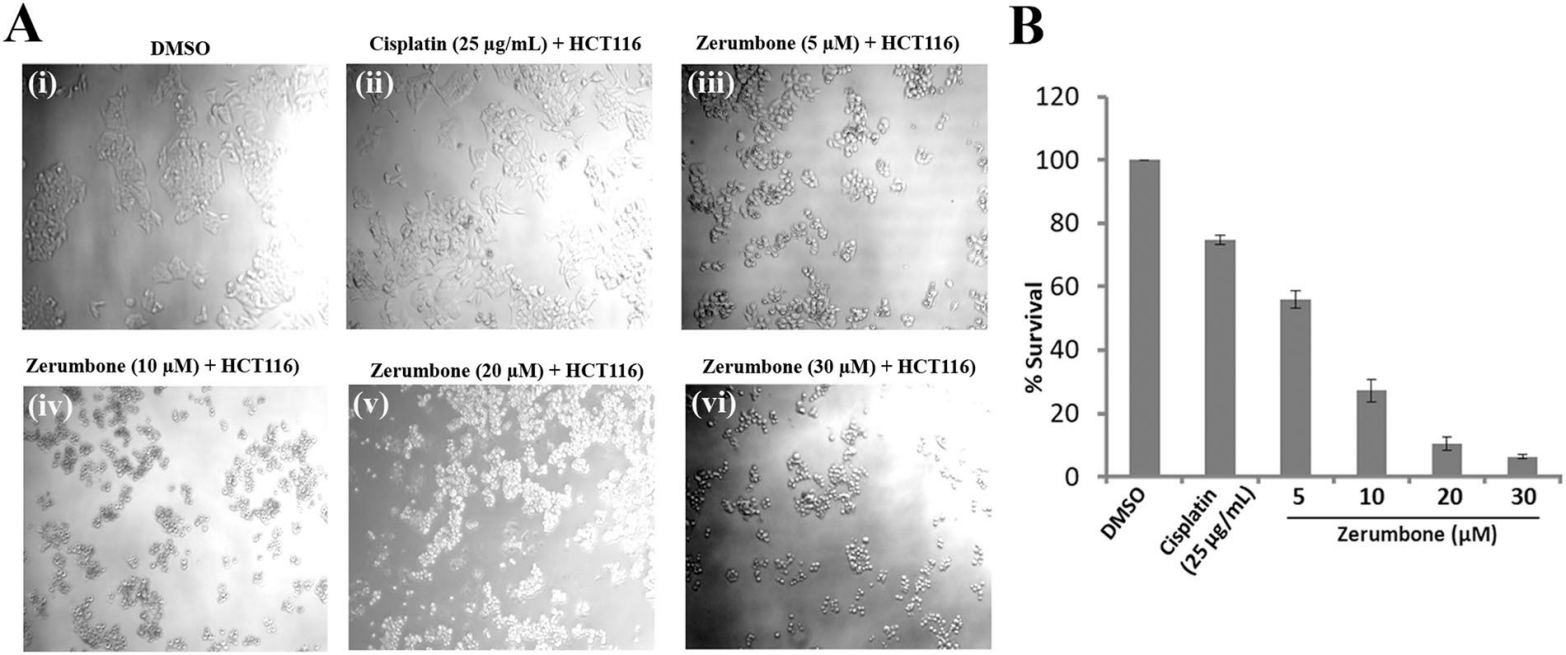
(**A**) Morphological changes of HCT116 cells treated with zerumbone (iii, iv, v & vi) and cisplatin (ii) after 24 h hours. Control cells are shown in photos (i) for 24 h. and (**B**) Graph showing the dose dependent inhibition of cell proliferation. HCT116 cells were treated either with DMSO or with the indicated concentration of zerumbone or cisplatin and the viable cells were measured with the Cell Titer 96 Proliferation Assay (Promega). The data represent the percentage growth compared with DMSO. The data represent the average of 3 independent experiments, with standard deviations.

The cell line assay of HCT116 observed the death of the cells after 24 hours of treatment with 20–30 μM zerumbone concentration (Fig. 1A,B). The cell lines snap also portrayed that the amount of dead cells in zerumbone (30 μM) treated cells (Fig. 1A(iv)) is more than the cells treated with cisplatin (Fig. 1A(ii)). The study also observed that 20–30 μM of zerumbone was adequate enough to inhibit the TNF-alpha with a higher TNF-alpha release value of 10.61 in case of 20 μM of zerumbone (Table 1). The positive control (cisplatin) has a lower TNF-alpha release value of 6.01 (Table 1) which implies zerumbone as good inhibitor than the known anti-cancer compound. However, zerumbone treated HCT116 in the concentration of 5 μM and 10 μM were unable to kill the cells with more than 35% survival rate in case of 10 μM and 56% survival rate in case of 5 μM zerumbone concentration (Fig. 1B). The TNF-alpha release value was 7.08 and 7.61 respectively for the zerumbone concentration 5 μM and 10 μM (Table 1). In fact, the activity of the zerumbone is because of the alpha-beta unsaturated carbonyl groups which are considered as an active moiety. Additionally, the dose-response curve and bar graph plot from the ELISA assay confirms that 20 μM of zerumbone was good enough to inhibit the TNF-alpha enzyme (Fig. SF2 A and B of supplementary material). Thus zerumbone was found to be active against HCT116 cells leading to apoptosis.

**Table 1:** ELISA assay for TNF-alpha inhibition.

SN	Compound and Concentration	TNFα inhibition ± SEM
1	Cisplatin (25 µg/µL)	6.01 ± 0.02
2	Zerumbone (2 μM)	5.80 ± 0.07
3	Zerumbone (5 μM)	7.08 ± 0.30
4	Zerumbone (10 μM)	7.61 ± 0.42
5	Zerumbone (15 μM)	8.42 ± 0.26
6	Zerumbone (20 μM)	10.05 ± 0.15

The docking score of the best top five conformational pose of zerumbone is presented in Table 2. Whereas the docking score of the best top five conformational pose α-humulene is presented in Table 3. In both the cases (Tables 2 and 3), the best pose was chosen based on the MolDock score, rerank score, interaction energy and binding affinity. The best pose of zerumbone (Pose No. 545) had an overall score of −138.41 kJ/mol (Table 2). While the best pose of α-humulene had an overall score of −121.78 kJ/mol (Table 3). Thus, indicating that zerumbone is a better inhibitor of TNF-alpha than α-humulene. Interestingly, the structure of zerumbone is 90% similar to α-humulene except that zerumbone has an extra alpha-beta unsaturated carbonyl group which makes zerumbone an active biomolecule. And this may be the reason for zerumbone possessing an overall favourable docking score than α-humulene at the active site of the TNF-alpha enzyme.

**Table 2:** Docking score of the top 5 docking poses of zerumbone.

SN	Docking Pose	MolDock Score	Rerank Score	Interaction Energy	Binding affinity	Overall Score
1	Pose No. 545	−45.46	−31.12	−44.01	−17.82	−138.41
2	Pose No. 968	−45.76	−31.13	−44.30	−16.66	−137.85
3	Pose No. 481	−44.63	−31.44	−43.18	−16.66	−135.91
4	Pose No. 97	−41.70	−31.69	−40.26	−16.66	−130.31
5	Pose No. 676	−38.61	−28.58	−37.15	−17.82	−122.16

**Table 3:** Docking score of the top 5 docking poses of α-humulene.

SN	Docking Pose	MolDock Score	Rerank Score	Interaction Energy	Binding affinity	Overall Score
1	Pose No. 616	−39.66	−29.17	−35.98	−16.97	−121.78
2	Pose No. 266	−38.13	−27.62	−34.45	−16.97	−117.17
3	Pose No. 436	−37.93	−26.41	−34.25	−16.98	−115.57
4	Pose No. 295	−37.15	−26.32	−33.47	−16.95	−113.89
5	Pose No. 385	−34.57	−25.30	−30.90	−16.97	−107.74

**Figure 2:**
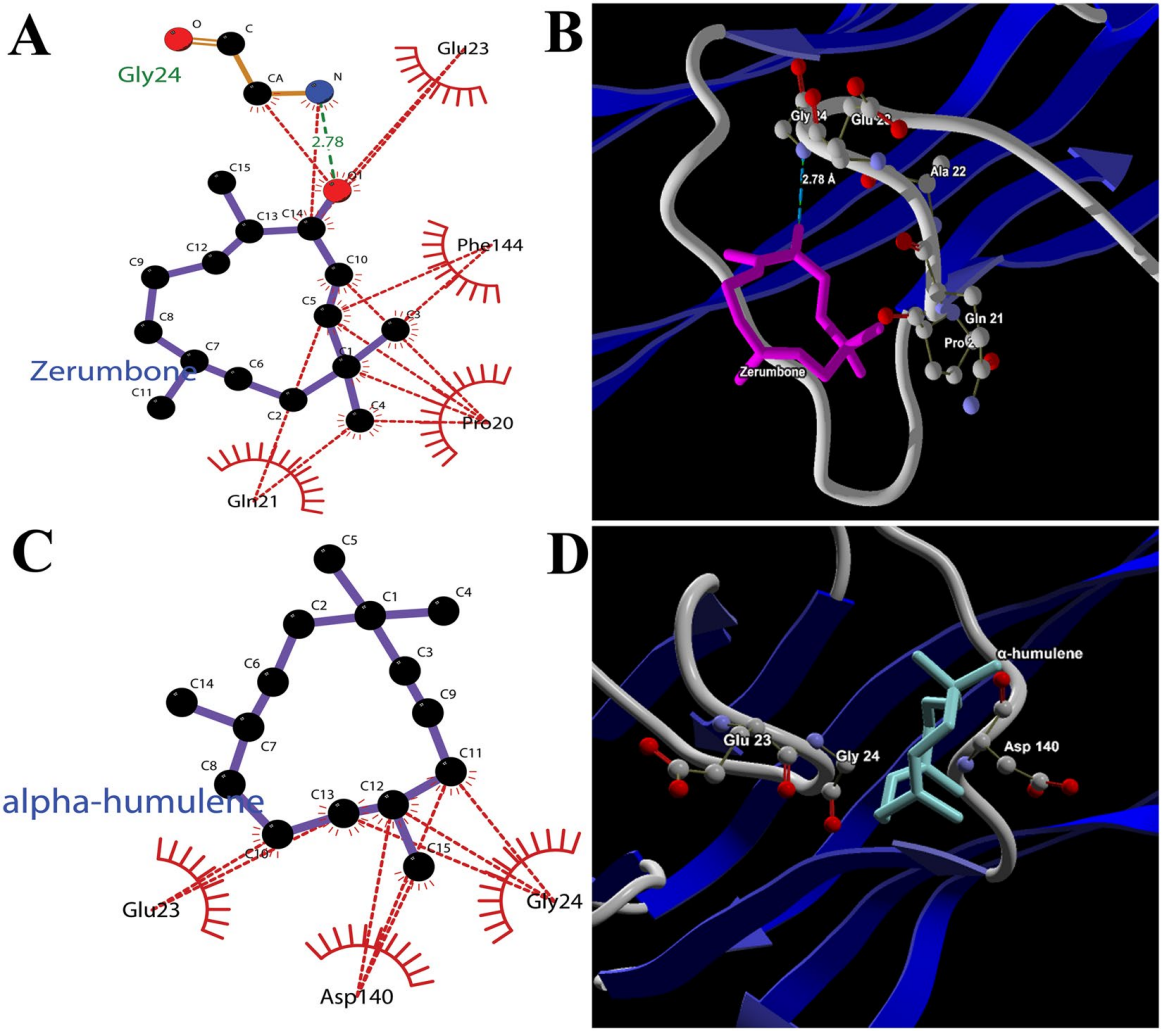
(**A**) 2D interaction map of zerumbone (**B**) 3D interaction map of zerumbone (**C**) 2D interaction map of α-humulene and (**D**) 3D interaction map of α-humulene at the active site of TNF-alpha enzyme (PDB ID: 5MU8). One hydrogen bond interaction (Gly24) and four hydrophobic interactions (Pro20, Gln21, Glu23 and Phe144) were observed in case of zerumbone with. Whereas only three hydrophobic interactions (Glu23, Glu24 and Asp140) was observed in case of α-humulene. Thus indicating a stronger binding force in zerumbone-TNF-alpha docked complex.

Figures 2A,B depicted the steric and hydrophobic interaction of zerumbone with Pro20, Gln21, Gly23, Gly24 and Phe144 residues of 5MU8. While Fig. 2C,D depicted the interaction of α-humulene with Glu23, Glu24 and Asp140. In case of TNF-alpha-zerumbone docked complex, a hydrogen bond interaction was observed with Gly24 whereas no hydrogen bonding was observed in TNF-alpha- α-humulene docked complex. Thus, suggesting a stronger binding affinity and hence a concrete molecular interaction in case of TNF-alpha-zerumbone docked complex.

The present report on the association of zerumbone and TNF-alpha using *in vitro* techniques is the first such report which is supported by computational studies. Moreover, the investigated enzyme is a TNF-α promotion signaling pathway that interferes with the proliferation of colon cancer cells. The strong interaction of zerumbone is because of the alpha-beta unsaturated carbonyl group which is a notable moiety that theater in certain unidentified targets and enzyme^28^. Similarly, Murakami *et al*. observed that the absence of alpha-beta unsaturated carbonyl group in compounds such as alpha-humulene, were unable to disrupt various tumour promoter enzymes such as 12-O-Tetradecanoylphorbol-13-acetate (TPA)^7^. In fact the difference in their structure lies in the carbonyl group present in zerumbone but not in alpha-humulene^29^. Thus, indicating that the alpha-beta unsaturated carbonyl group is the key component of zerumbone for its biological activity against cancer cell lines and protein targets^30^. The overall score revealed favourable binding energy and strong bonding which were confirmed from the docking scores with the TNF-α enzyme (Tables 2 and 3).

**Figure 3:**
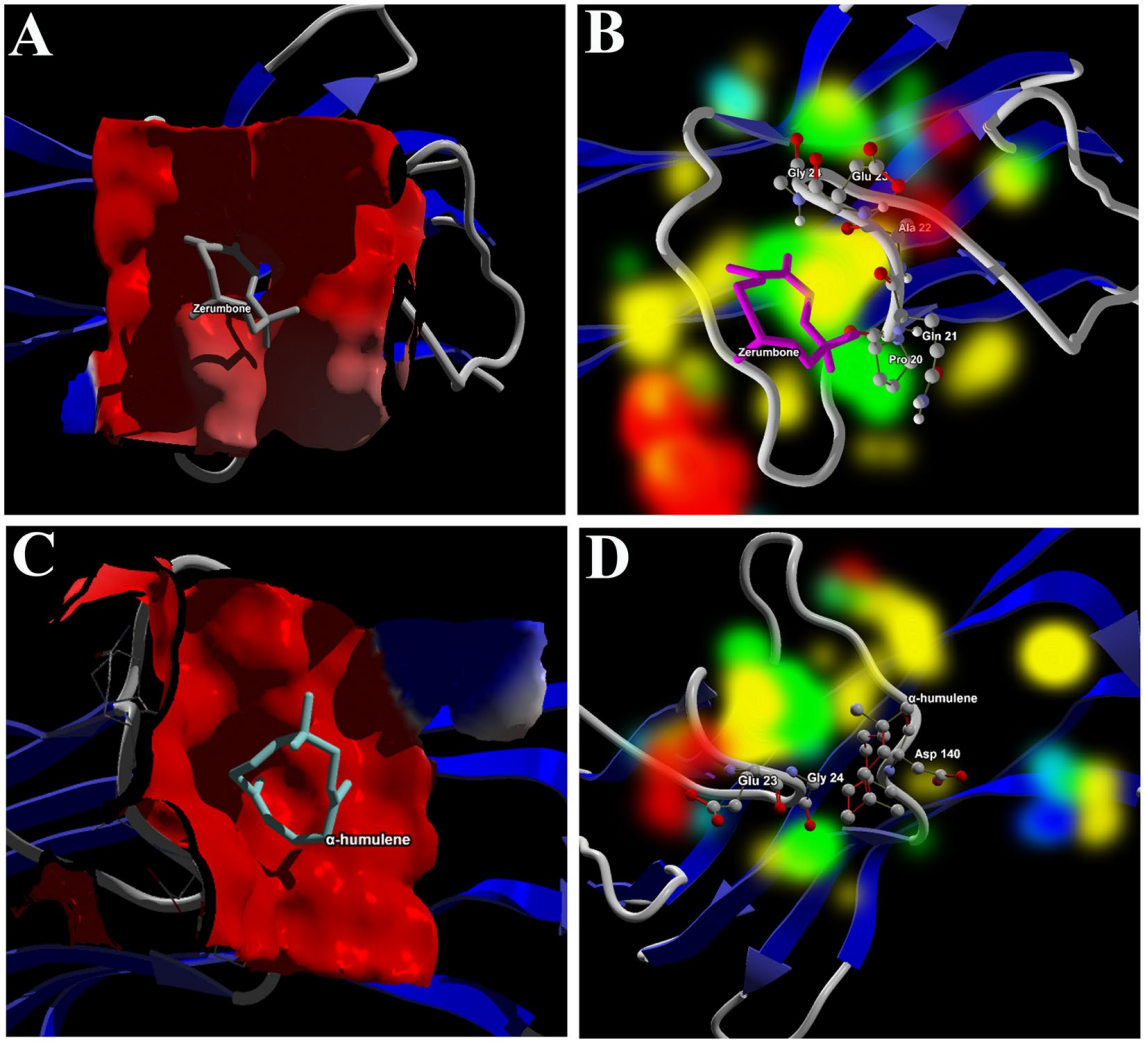
Electrostatic interaction map of (**A**) zerumbone and (**C**) α-humulene. Energy map of (**B**) zerumbone and (**D**) α-humulene at the active site of TNF-alpha enzyme. Figure 3A depicted zerumbone lying inside the surface of the Electrostatic pocket whereas in Fig. 3B, α-humulene lies above the surface of electrostatic pocket.

Figure 3 depicts the electrostatic interaction map and energy map of the best docking pose of zerumbone and α-humulene at the binding site of the TNF-alpha (5MU8). It is evident from both the maps that zerumbone possessed fair affinity of energy based interactions compared to α-humulene. As depicted in Fig. 3A,C, zerumbone lies inside the surface of the electrostatic pocket whereas in α-humulene lies above the surface of the electrostatic pocket. Moreover, the 3D hydrophobic interaction map of zerumbone and α-humulene observed that zerumbone lies closer to the hydrophobic pocket compared to α-humulene (Figs SF3 and SF4 of supplementary material)

Additionally, zerumbone is surrounded by a pool of energy based favourable interactions such as hydrogen bond donor/acceptor favourable regions, electrostatic favourable and steric favourable regions (Fig. 3B). However, there are no experimental data available for zerumbone binding to TNF-alpha enzyme based on the available reports. Thus, there is a fair affinity between zerumbone and TNF-alpha.

**Figure 4:**
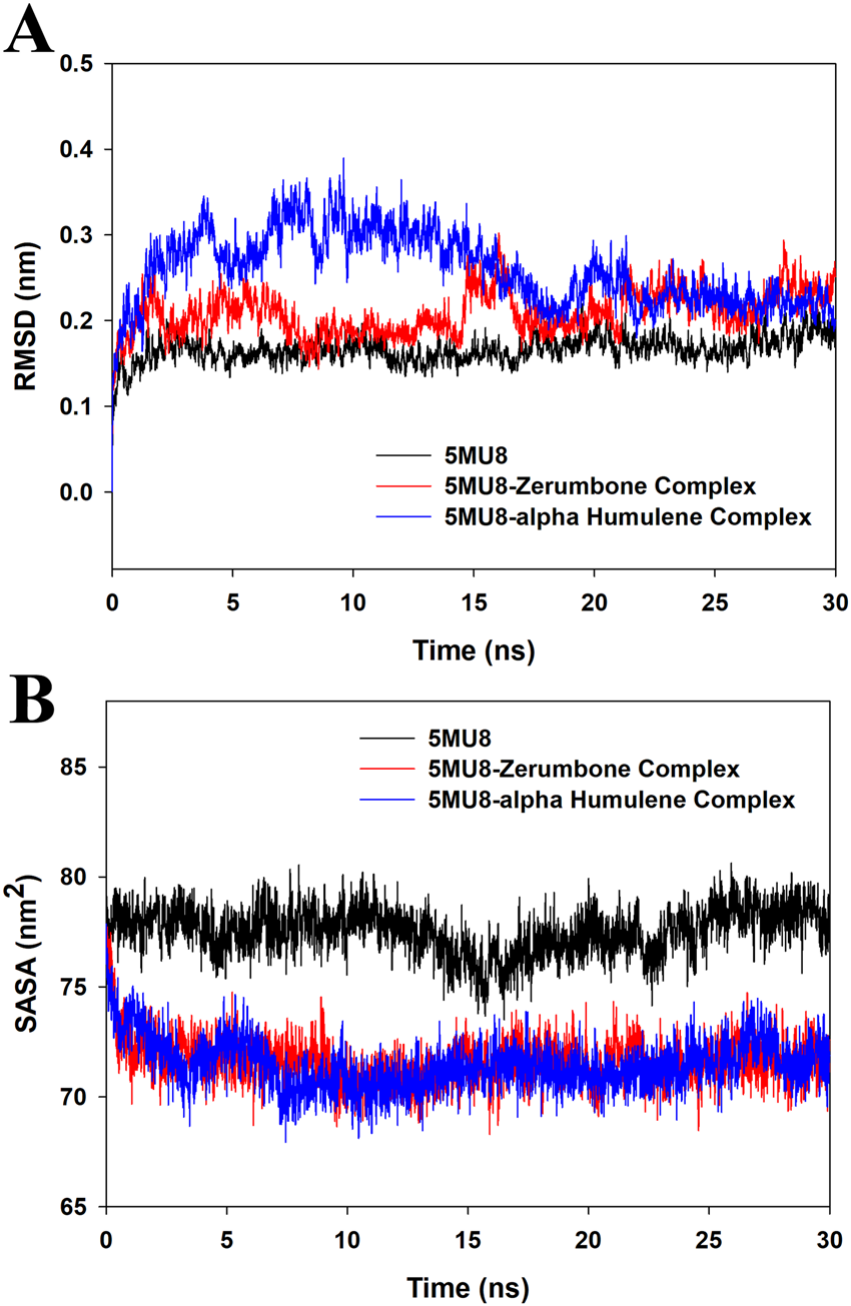
Trajectories from the MD Simulation production representing (**A**) RMSD (root mean squared deviation) and (**B**) Radius of gyration of the TNF-alpha and TNF-alpha-zerumbone and α-humulene docked complex.

**Figure 5:**
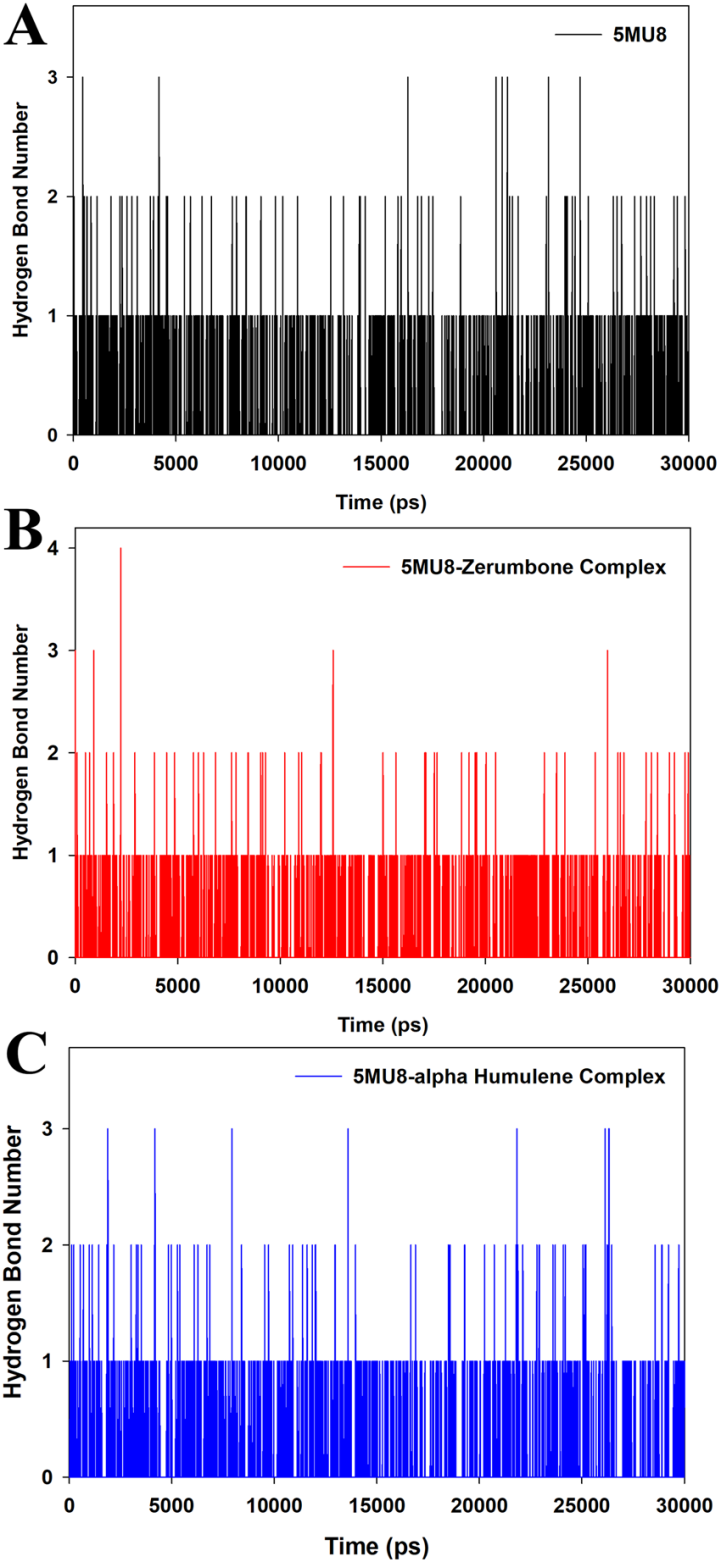
Hbond interaction number of (**A**) TNF-aplha (**B**) TNF-alpha-zerumbone and (**C**) α-humulene docked complex.

Figure 4A depicts the RMSD graph processed from the trajectory file of the 30 ns MD production. The 5MU8-zerumbbone complex showed a minor shift at 15 ns, but a stable conformation in the preceding MD production till 30 ns. However, the 5MU8-α-humulene complex observed a major shift and deviations in the first 20 ns MD production as evident from the RMSD graph (Fig. 4A). Hence, the 5MU8-zerumbbone docked complex confirms a stable conformation. On the other hand, Fig. 4B depicts the SASA energy analysis where the graph depicted a uniform hydrophobicity interaction of the protein-ligand complexes during the 30 ns run. However, there were not many variations in the RMSF values and R*g* graph between the two zerumbone and α-humulene complexes (Figs SF5 and SF6 of supplementary material). While Figs. 5A, B and C represent the measurement of the hydrogen bond distances of the 5MU8, 5MU8-zerumbone and 5MU8-α-humulene docked complexes. Figure 5B confirms that the docked complex have more hydrogen bond interaction raising up to four numbers thus showing a stable system and a good hydrogen bond interaction.

The binding free energy calculation of the protein-ligand interaction from the 100 snapshots extracted every 100 ps from the last 30 ns MD simulation stable trajectory is shown in Table 4. The binding free-energy (ΔG_*bind*_) values of the 5MU8-zerumbone complex and 5MU8-α-humulene complex were −52.10 kJ/mol and −40.23 kJ/mol respectively. The ΔG_*bind*_ values suggest that 5MU8-zerumbone complex have better binding free-energy than the 5MU8-α-humulene complex.

**Table 4:** Binding free-energy (ΔG_bind_) of TNF-alpha-zerumbone and TNF-alpha-α-humulene docked complex calculated using g_mmpbsa.

Energy parameters (kJ/mol)	Zerumbone (kJ/mol)	α-humulene (kJ/mol)
van der Waal energy	−66.06 ± 43.07	−53.12 ± 33.12
Electrostatic energy	−11.80 ± 16.70	−8.12 ± 11.45
Polar solvation energy	31.46 ± 34.61	24.13 ± 27.32
SASA energy	−5.70 ± 3.80	−3.12 ± 2.21
Binding energy	−52.10 ± 38.67	−40.23 ± 34.12

In Table 3, the van der Waals energy and electrostatic energy was found higher for the zerumbone complex than α-humulene complex. As it has been seen earlier in docking and interaction analysis study, that zerumbone formed hydrogen bond interaction with Gly24 whereas α-humulene does not form any hydrogen bond interaction contact (Fig. 2C,D). The 5MU8-zerumbone complex proves to be stronger than 5MU8-α-humulene complex. In fact, the hydrogen bond interaction also played an important role in stabilizing the complexes energetically. Overall, all the energy parameters showed significantly favourable in the zerumbone complex thus indicating a favourable binding free-energy than α-humulene complex.

Moreover, the electrostatic energy and SASA energy were also favourable for the binding. These results also suggested that hydrophobic interaction played an important role in protein-ligand binding.

**Figure 6:**
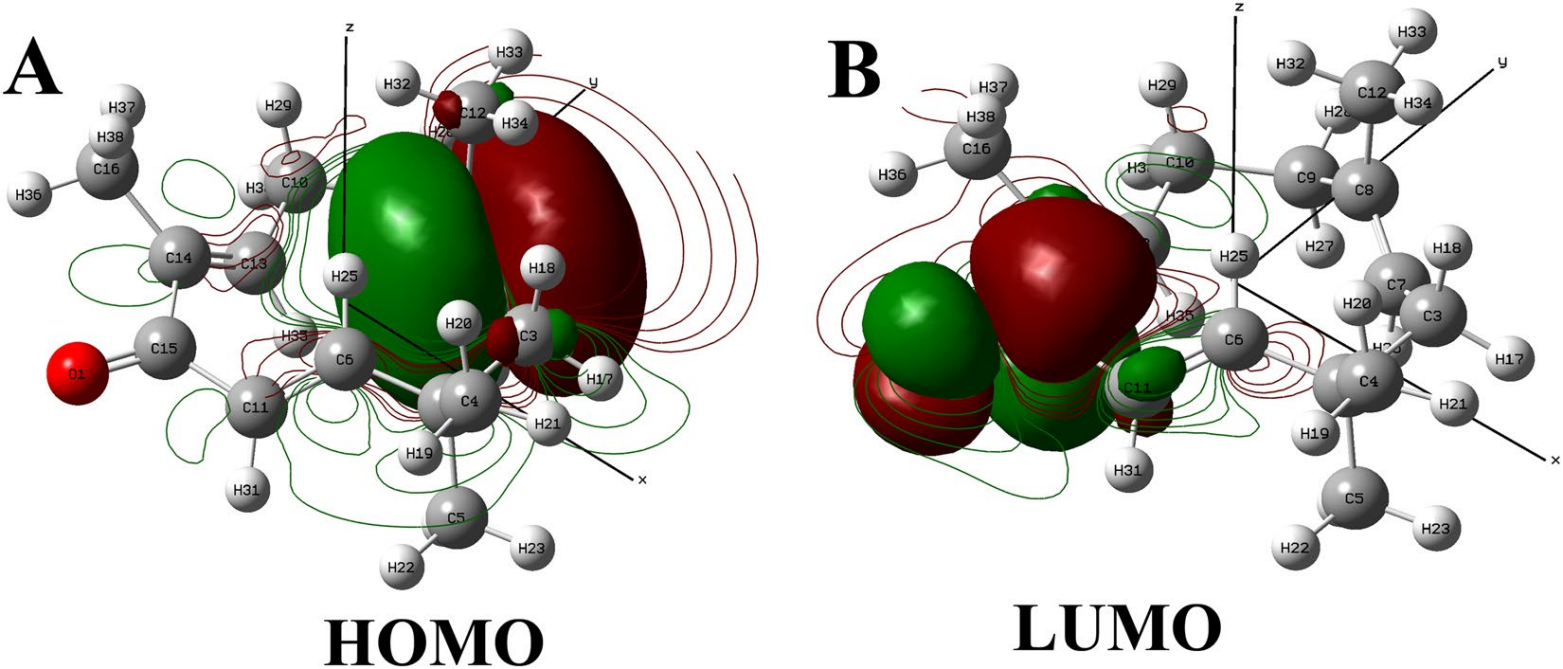
Contour map depicting the (**A**) HOMO and (**A**) LUMO orbital energies of zerumbone calculated at DFT/B3LYP/ LanL2DLZ level of theory.

**Figure 7:**
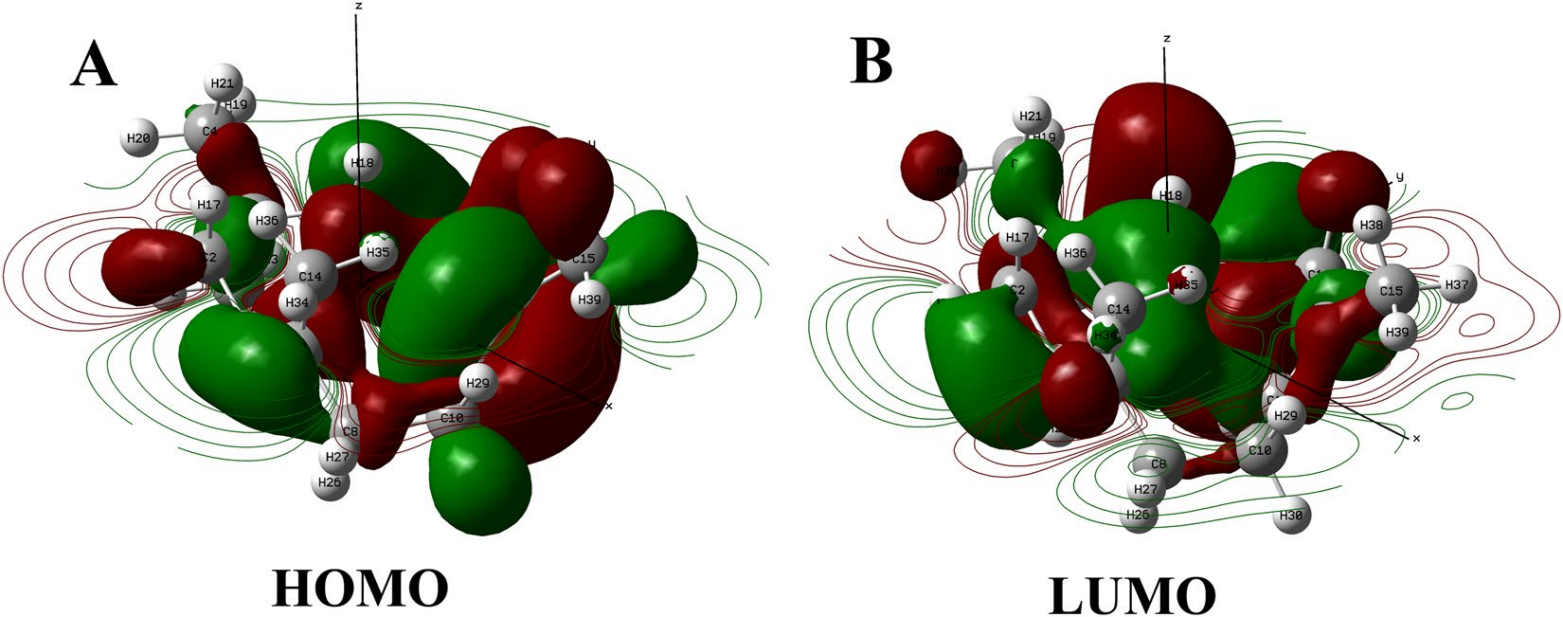
Contour map depicting the (**A**) HOMO and (**A**) LUMO orbital energies of α-humulene calculated at DFT/B3LYP/ LanL2DLZ level of theory.

Figures 6 and 7 depict the HOMO and LUMO energies of the best docked pose of zerumbone and α-humulene. While The HOMO and LUMO energies aid in understanding the band gap energy of the docked pose. A low band gap indicates a higher reactivity and thus the compound is believed to be strong enough to bind at the protein’s active site. The band gap energy was (ΔE_*LUMO - HOMO*_) was −0.12 eV and −0.08 eV for zerumbone and α-humulene which confirms that zerumbone had a lower band gap and will definitely have a strong binding affinity at the active site of TNF-alpha (5MU8). Additionally, the predicted NMR and IR spectra of zerumbone and α-humulene which optimized at DFT/B3LYP/LanL2DLZ level of theory are presented in Figs SF7–SF10 in supplementary material

**Figure 8:**
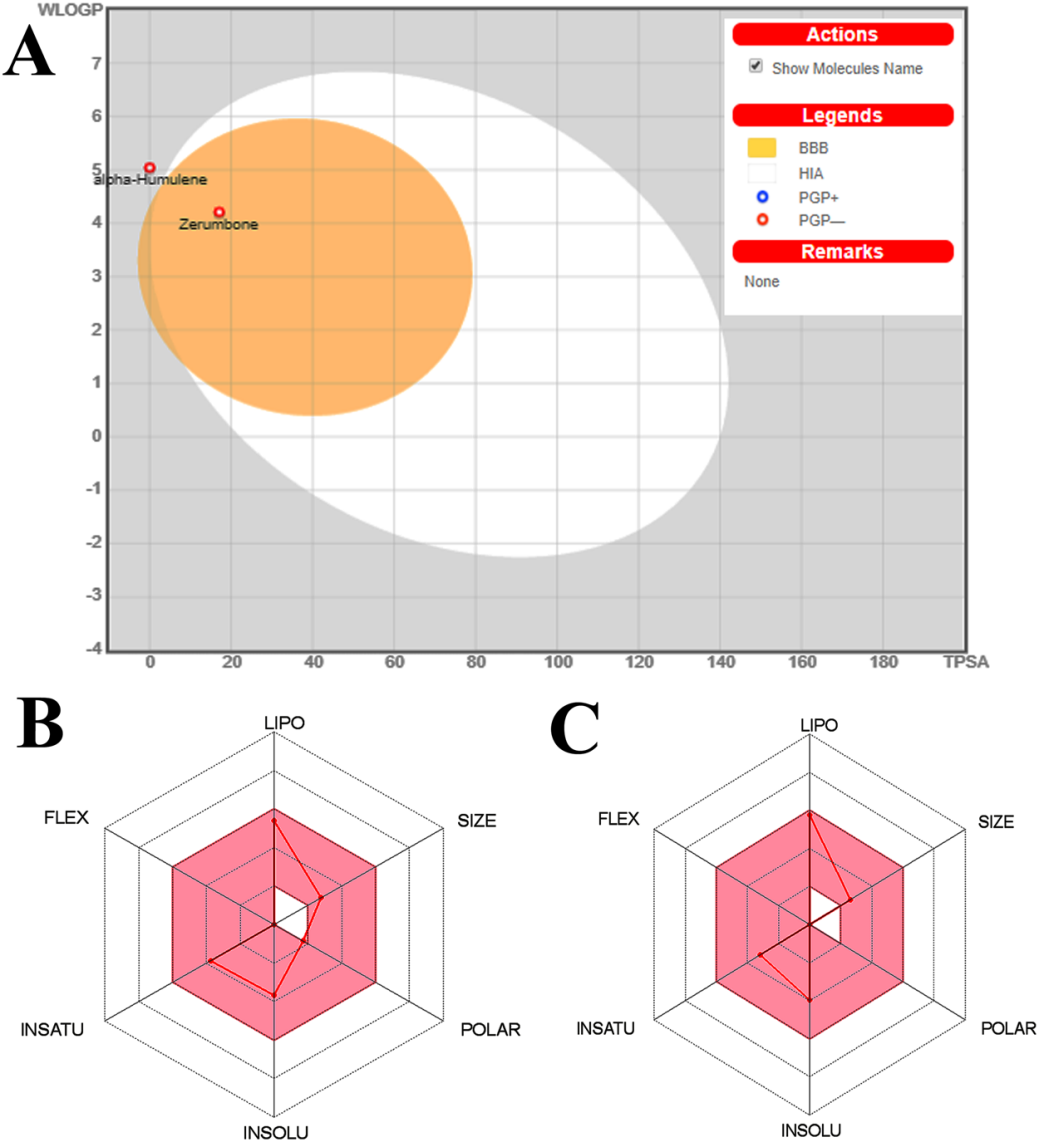
(**A**) Boiled egg diagram of zerumbone and α-humulene and Bioavailability Radar map of depicting the LIPO (lipophilicity), SIZE (molecular weight), POLAR (pplarity), INSOLU (insolubility) INSATU (insaturation) and FLEX (rotatable bond flexibility) parameters of (**B**) zerumbone and (**C**) α-humulene.

**Figure 9:**
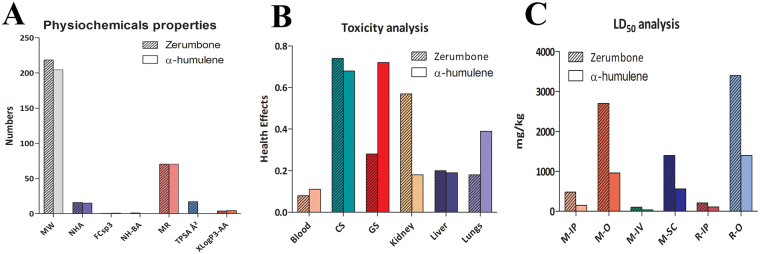
(**A**) Physiochemical properties of zerumbone and α-humulene representing the molecular weight (MW), number of heavy atoms (NHA); fraction Csp3 (FCsp3); number of H-bond acceptors (NH-BA); molar refractivity (MR), topological polar surface area (TPSA) and XLogP3-AA. (**B**) Toxicity analysis of zerumbone and α-humulene on blood, cardiovascular system (CS), gastrointestinal system (GS), kidney, liver and lungs. (**C**) LD_50_ analysis of Zerumbone and α-humulene on Mouse/Intraperitoneal (M-IP); Mouse/Oral (M-O); Mouse/Intravenous (M-IV); Mouse/Subcutaneous (M-SC); Rat/Intraperitoneal (R-IP) and Rat/Oral (R-O).

The Boiled egg diagram and bioavailability radar map of zerumbone and α-humulene are presented in Fig. 8. As evident from the Fig. 8 zerumbone has a better oral bioavailability than α-humulene. The physiochemical property analysis also revealed that zerumbone had TPSA of 17.07 Å² compared to 0.00 Å² of α-humulene which indicates zerumbone is a good enzyme inhibitor (Table 5 and Fig. 9A). Table 6 revealed that zerumbone and α-humulene having similar behaviour of ADME parameters with a similar rate of absorption and Blood-Brain Barrier. Figure 9B and Table ST1 of supplementary observed that zerumbone is likely to have some chance of toxic effect on kidney and cardiovascular system. However, Fig. 9C and Table 7 confirms that the LD_50_ of zerumbone is much higher than α-humulene.

**Table 5:** Physiochemical properties of zerumbone and α-humulene.

Properties	Zerumbone	α-humulene
2D Structure		
Formula	C15H22O	C15H24
Molecular weight (MW)	218.33 g/mol	204.35 g/mol
Num. heavy atoms (NHA)	16	15
Num. arom. heavy atoms (NAHA)	0	0
Fraction Csp3 (FCsp3)	0.53	0.60
Num. H-bond acceptors (NH-BA)	1	0
Num. H-bond donors (NH-BD)	0	0
Molar Refractivity (MR)	70.62	70.42
Topological Polar Surface Area (TPSA)	17.07 Å²	0.00 Å²
XLogP3-AA	3.9	4.5

**Table 6:** ADME parameters of zerumbone and α-humulene computed using ACD iLab.

ADME parameters	Zerumbone	α-humulene
Absorption	8.43 × 10^−4^ cm/s	8.46 × 10^−4^ cm/s
Absorption rate K_a_	0.058 min^−1^	0.058 min^−1^
Blood-Brain Barrier (BBB)		
LogPS	−1	−1
LogPB	0.37	0.35
Log(PS*fu, brain)	−2.9	−3.2
LogBB	0.37	0.35
LogPS	−1	−1
DBP	96.92%	98.59%
Vd	2.23 L/kg	3.71 L/kg
Pgp Substrate	Non-substrate	Non-substrate
Pgp Inhibitor	Non-inhibitor	Non-inhibitor

LogPS: Rate of brain penetration; LogPB: Extent of brain penetration; Log(PS*fu, brain): Brain/plasma equilibration rate; LogBB: Blood-Brain Distribution (rodent); LogPS: BBB Permeability (rat); DBP: Drug binding to plasma proteins (human); Vd: Voume of Distrubtutin (human); Pgp Substrate: P-glycoprotein substrate specificity; Pgp Inhibitor: P-glycoprotein inhibitor specificity.

**Table 7:** LD50 analysis of Zerumbone and α-humulene.

LD_50_ Species /Administration route	LD_50_ of Zerumbone (mg/kg)	Reliability	LD_50_ of α-humulene (mg/kg)	Reliability
Mouse/Intraperitoneal	480	Moderate(0.66)	150	Borderline(0.37)
Mouse/Oral	2700	Moderate(0.51)	960	Borderline(0.38)
Mouse/Intravenous	100	Moderate(0.51)	34	Not Reliable(0.27)
Mouse/Subcutaneous	1400	Borderline(0.44)	560	Moderate(0.55)
Rat/Intraperitoneal	210	Borderline(0.38)	110	Borderline(0.44)
Rat/Oral	3400	High(0.8)	1400	Borderline(0.44)

## Conclusions

The present investigation confirms the inhibition of TNF-alpha by zerumbone and the decrease in HCT116 Cell proliferation in controlling colon cancer. The molecular docking study also confirms the binding mode of zerumbone at the active site of the enzyme. Moreover, the computational investigation such as the binding free-energy analysis on zerumbone and α-humulene point out that the presence of the alpha-beta unsaturated carbonyl group is a major driving force. Because, the compound α-humulene which lacks the alpha-beta unsaturated carbonyl group proved to be a bad inhibitor from the computational investigation as well as from the physiochemical property analysis and ADME-Toxicity analysis. In fact, the strong binding affinity and favourable docking score observed in zerumbone is because of the alpha-beta unsaturated carbonyl group which is also responsible for apoptosis. The 30 ns MD simulation confirmed the conformational stability based on the trajectory analysis and the DFT analysis confirms the reactivity nature of zerumbone based on the frontier molecular orbital analysis. Additionally, the physiochemical property and ADME-Toxicity analysis also confirms that it has good oral bioavailability and higher LD_50_.

## Electronic supplementary material

Supplementary Information
