# Supplementary material for: Zerumbone reduces proliferation of HCT116 colon cancer cells by inhibition of TNF-alpha
Source: Sci Rep. 2018 Mar 6;8:4090. doi: 10.1038/s41598-018-22362-1 (PMC5840388; doi:10.1038/s41598-018-22362-1)
Supplement: Supplementary file 1 — Supplementary Information [file 41598_2018_22362_MOESM1_ESM.docx]

**Supplementary material**

**Zerumbone reduces proliferation of HCT116 colon cancer cells by inhibition of TNF-alpha**

Salam Pradeep Singh^a^, Khumukcham Nongalleima^a^, Ningthoujam Indrajit Singh^a^, Pradip Doley^a^,

Chingakham Brajakisor Singh^a^*, Thiyam Ramsing Singh^b^ and Dinabandhu Sahoo^a^

aInstitute of Bio-resources and Sustainable Development, Takyelpat, Imphal-79500, Manipur,

India

bDepartment of Biotechnology, Manipur University, Canchipur-795003, Manipur.

*Corresponding author: [kishore.ibsd@nic.gov.in](mailto:kishore.ibsd@nic.gov.in)

**FIGURES**


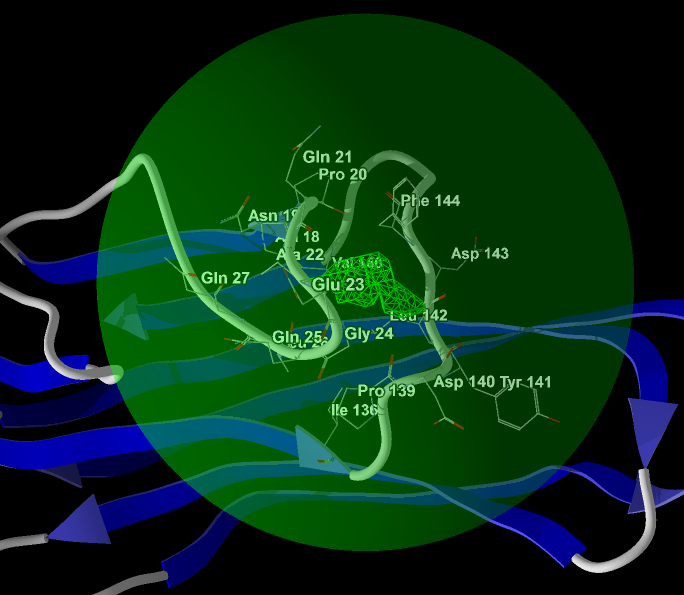


Fig. SF1. Binding cavity of TNF-alpha (PDB ID: 5MU8)


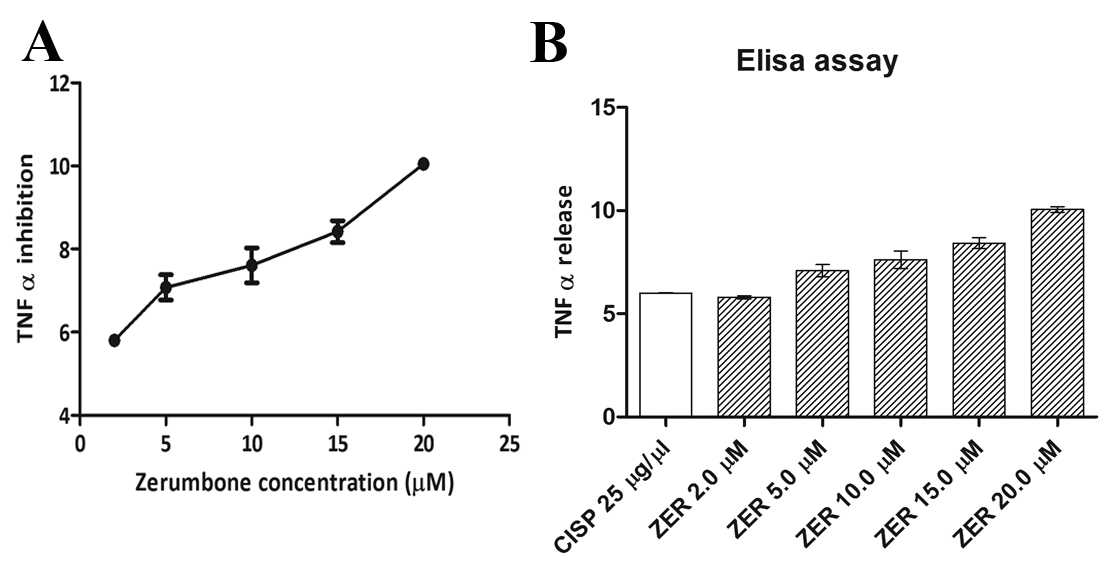


Fig. SF2. (A) Dose response curve and (B) ELISA assay of HCT116 cells treated with various concentrations of zerumbone (ZER) (2 µM, 5 µM, 10 µM, 15 µM and 20 µM) for 24 h and cisplatin (CISP). Error bars represent standard error.


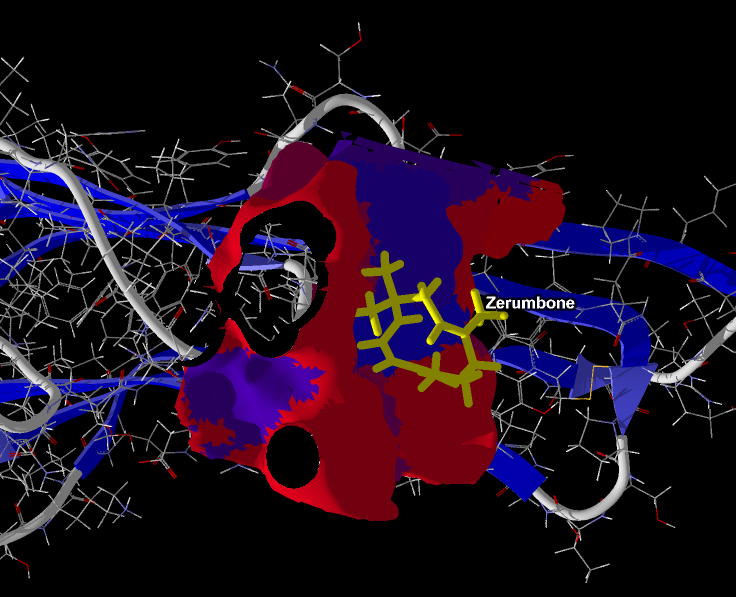


Fig. SF3. Hydrophobic interaction map of zerumbone at the active site of TNF-alpha


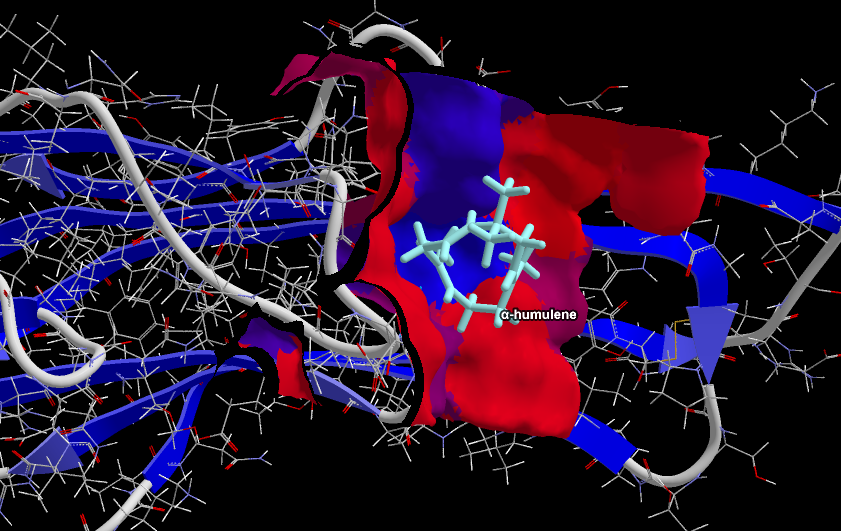


Fig. SF4. Hydrophobic interaction map of alpha humulene at the active site of TNF-alpha


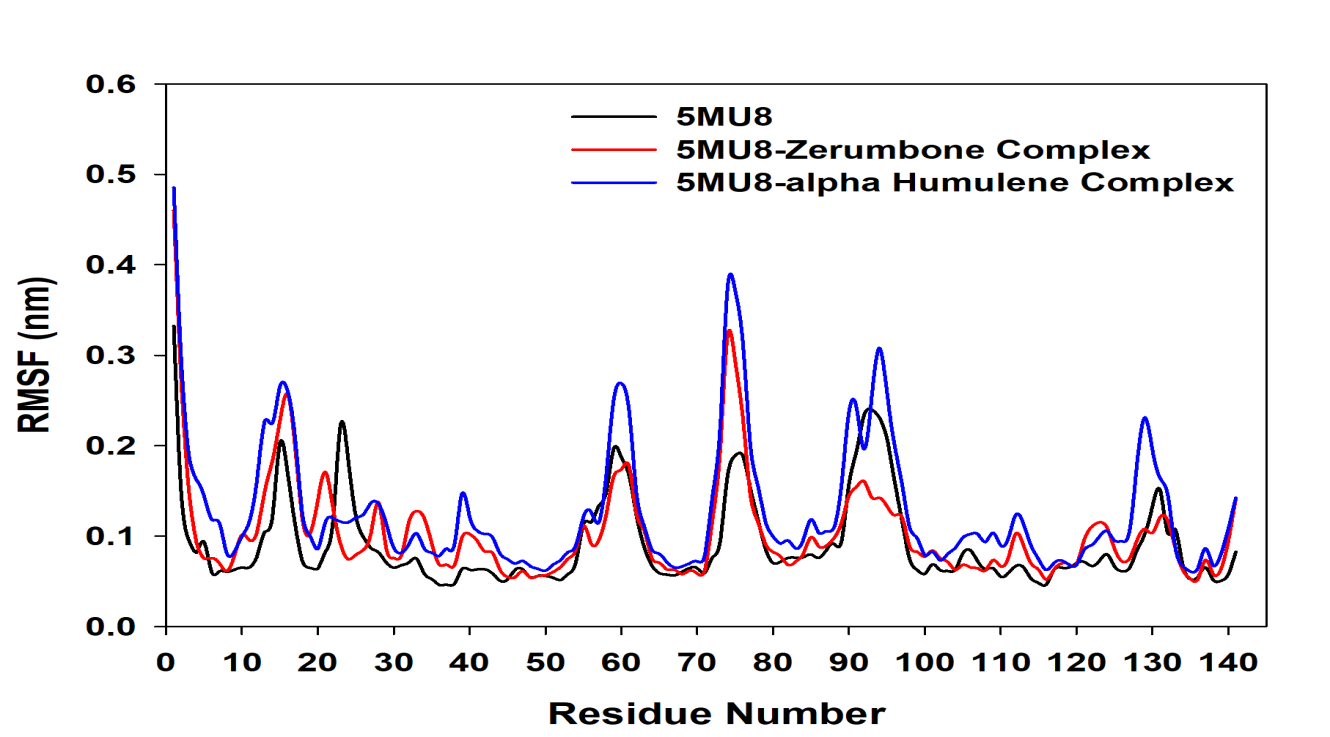


Fig. SF5. Trajectories from the MD Simulation production representing the RMSF of the TNF-alpha (5MU8), 5MU8-zerumbone and 5MU8-α-humulene docked complex


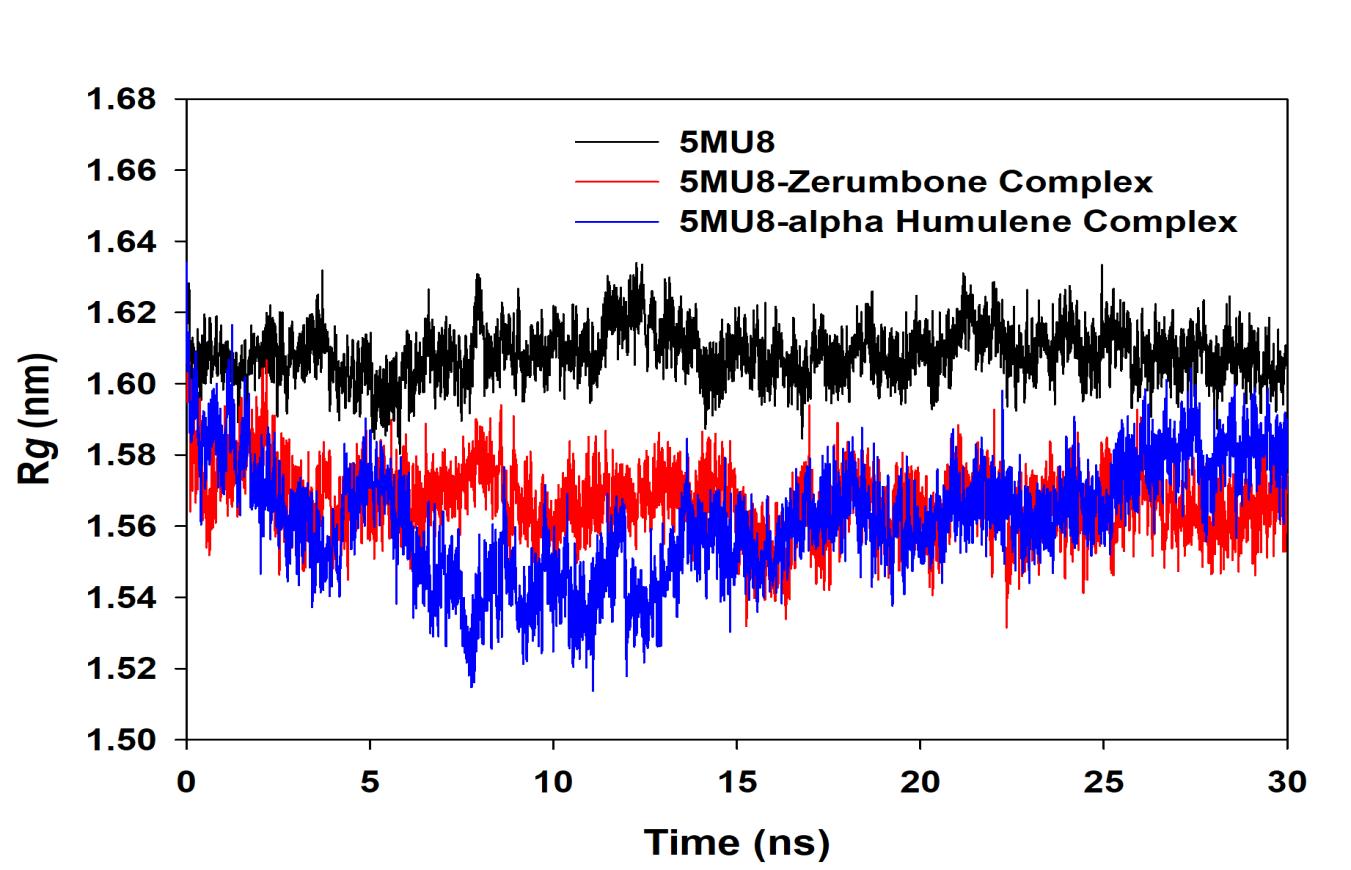


Fig. SF6. Trajectories from the MD Simulation production representing the radius of Gyration (R*g*) of the TNF-alpha (5MU8), 5MU8-zerumbone and 5MU8-α-humulene docked complex


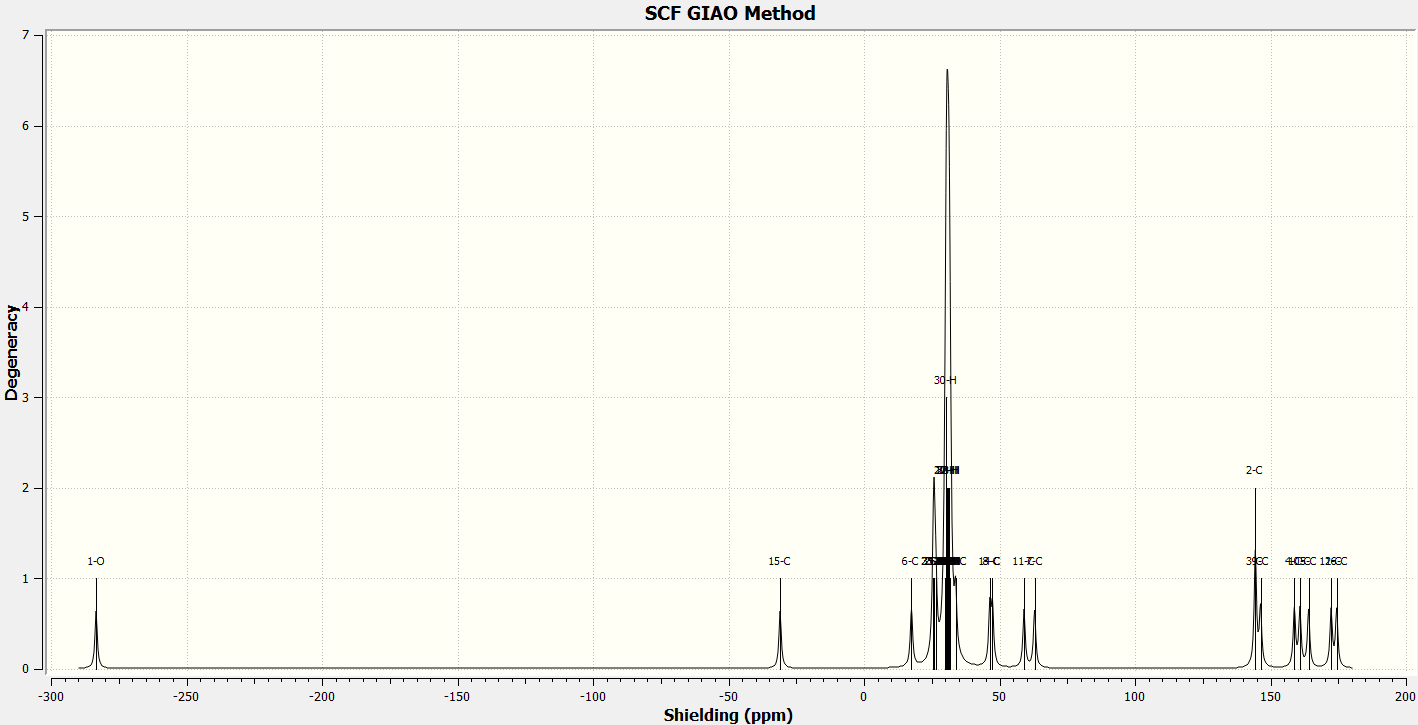


Fig. SF7. Predicted NMR spectra of zerumbone optimized at DFT/B3LYP/LanL2DLZ level of theory


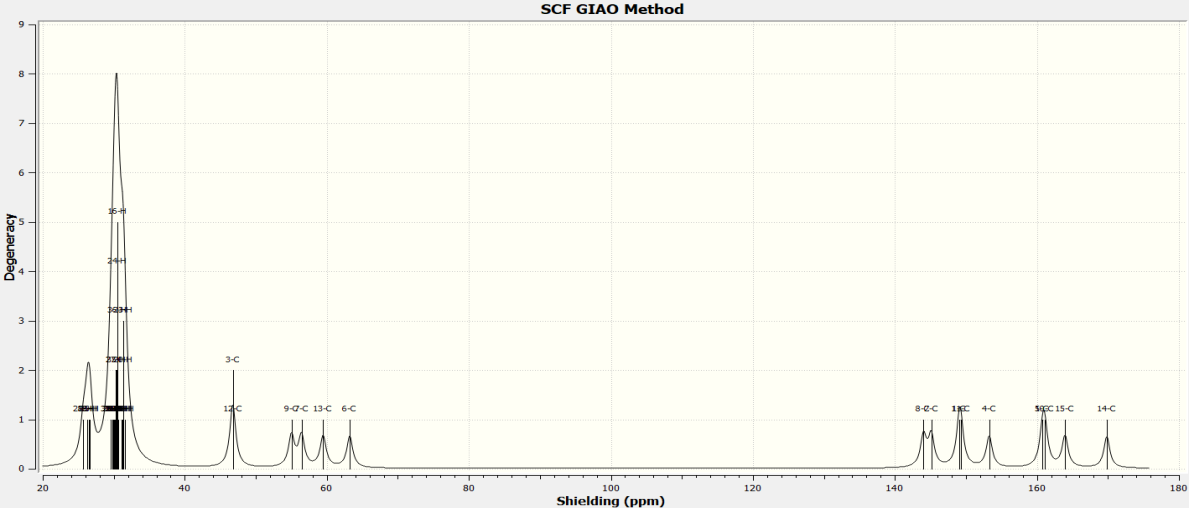


Fig. SF8. Predicted NMR spectra of alpha humulene optimized at DFT/B3LYP/LanL2DLZ level of theory


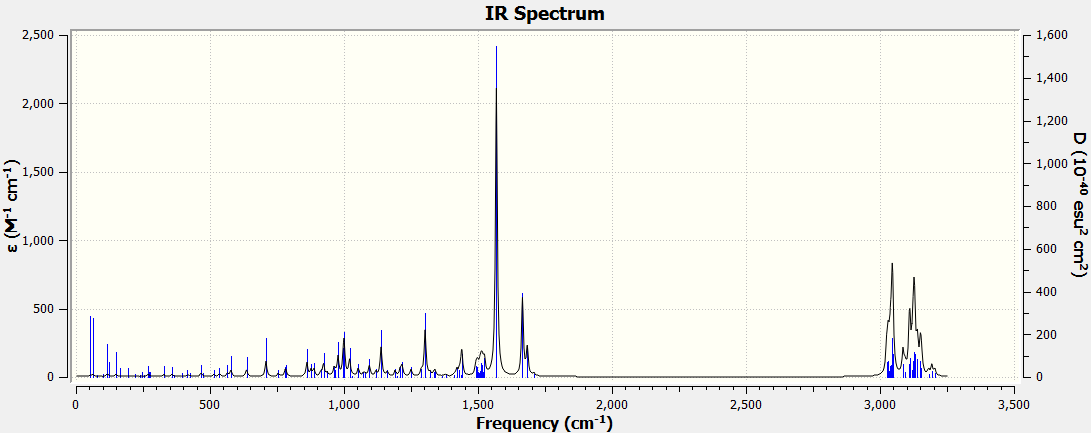


Fig. SF9. Predicted IR Spectra of zerumbone optimized at DFT/B3LYP/LanL2DLZ level of theory


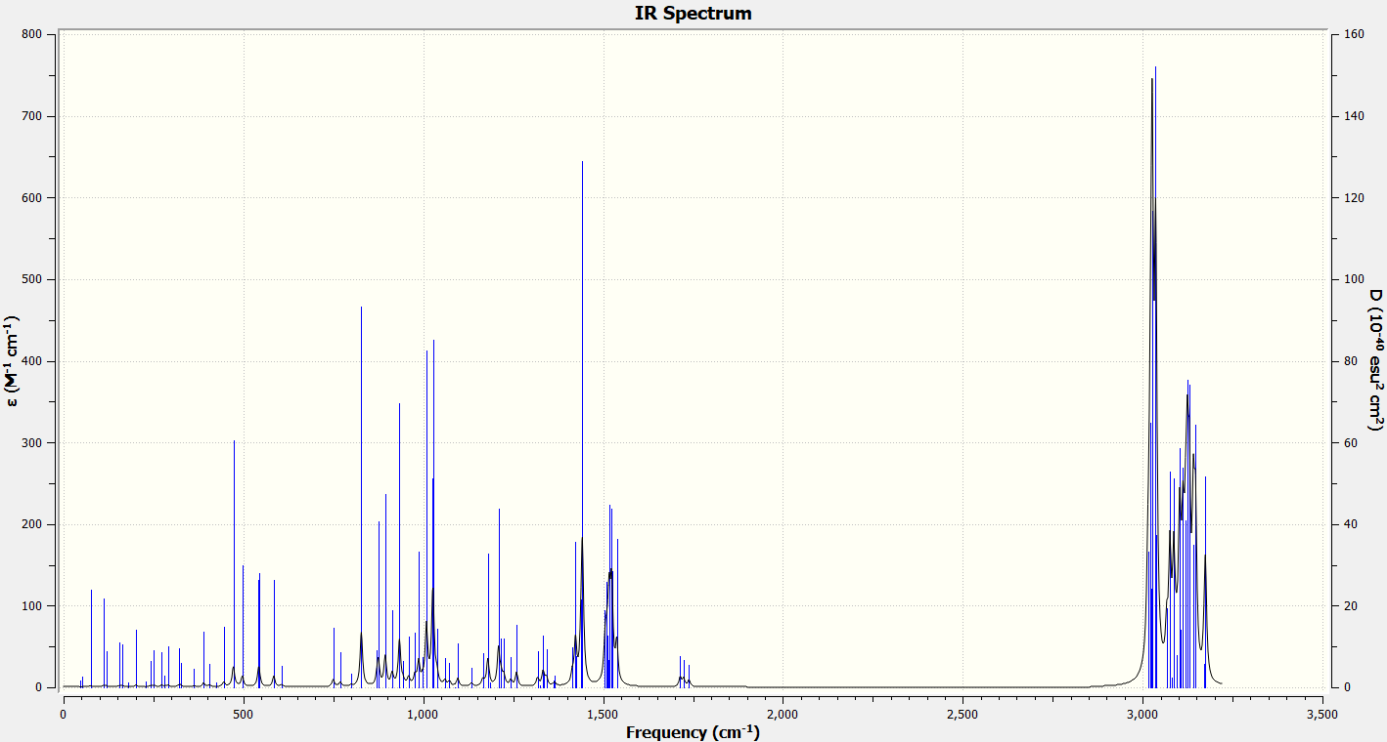


Fig. SF10. Predicted IR Spectra of alpha humulene optimized at DFT/B3LYP/LanL2DLZ level of theory

**TABLES**

**Table ST1. Toxicity analysis of zerumbone and α-humulene on various organ and systems**

| **Toxic health effects on** | **Zerumbone** | **Probable Atomic position** | **α-humulene** | **Probable Atomic position** |
| --- | --- | --- | --- | --- |
| Blood | **0.08** | 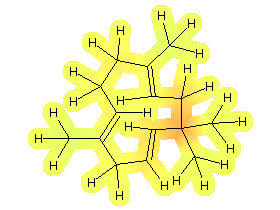 | **0.11** | 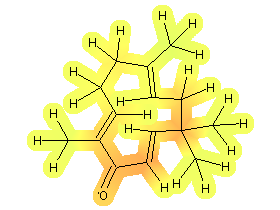 |
| Cardiovascular system | **0.74** | 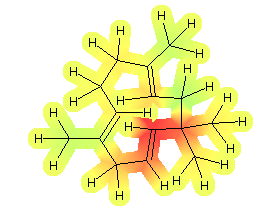 | **0.68** | 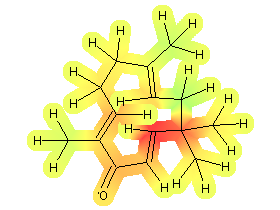 |
| Gastrointestinal system | **0.28** | 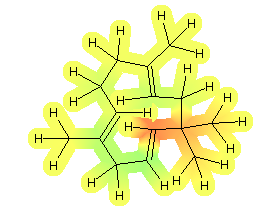 | **0.72** | 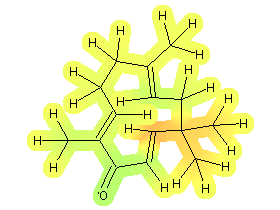 |
| Kidney | **0.57** | 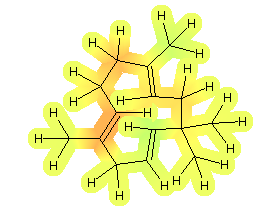 | **0.18** | 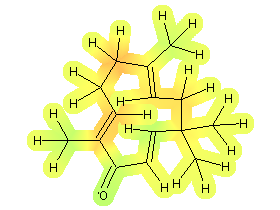 |
| Liver | **0.2** | 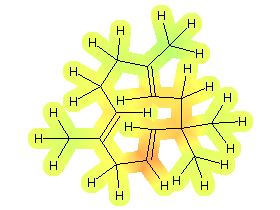 | **0.19** | 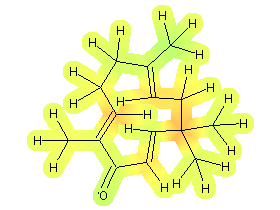 |
| Lungs | **0.18** | 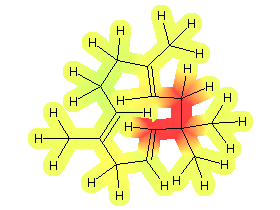 | **0.39** | 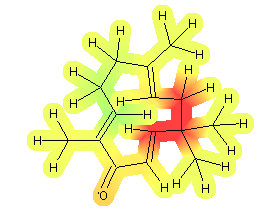 |
